# Le syndrome de Heerfordt

**DOI:** 10.11604/pamj.2015.22.307.7515

**Published:** 2015-11-26

**Authors:** Nabil Hammoune, Hicham Janah

**Affiliations:** 1 Service de Radiologie, 3ème Hôpital Militaire Laayoune, Maroc; 2 Service de Pneumologie, 3ème Hôpital Militaire Laayoune, Maroc

**Keywords:** Sarcoïdose, parotidite, uvéite, Sarcoidosis, parotitis, uveitis

## Image en medicine

La sarcoïdose est une granulomatose systémique de cause inconnue, caractérisée par son polymorphisme clinique et une grande variété de ses modes de présentation. L'association de fièvre, d'uvéite, de parotidite et d'une paralysie faciale périphérique réalise le syndrome de Heerfordt qui présente une manifestation inhabituelle révélatrice de cette maladie. Nous rapportons le cas d'un patient de 46 ans qui présentait depuis trois mois une parotidomégalie bilatérale indolore et une fièvre (A). L'examen général était sans anomalies en particulier l'absence de paralysie faciale, la biologie était normale. L'échographie cervicale montrait une hypertrophie parotidienne bilatérale d'échostructure hétérogène multinodulaire en rapport avec des multiples images nodulaires hypoéchogènes de taille variable sans adénopathie cervicale (B) , l'examen ophtalmologique révélait une uvéite postérieure bilatérale associée à des nodules choroïdiens, la radiographie et le scanner thoracique révélaient des adénopathies médiastinales et une atteinte pulmonaire micronodulaire (C), l'intradermoréaction à la tuberculine était négative, le diagnostic de sarcoïdose était alors évoqué et confirmé par la biopsie des parotides, le patient a été mis sous corticothérapie par voie générale ce qui a permis la régression de la symptomatologie. Le syndrome de Heerfordt est une manifestation clinique inhabituelle (moins de 6%) au cours d'une sarcoïdose systémique, caractérisée par la triade parotidite, uvéite et paralysie faciale périphérique. Le traitement de choix est la corticothérapie par voie générale.

**Figure 1:**
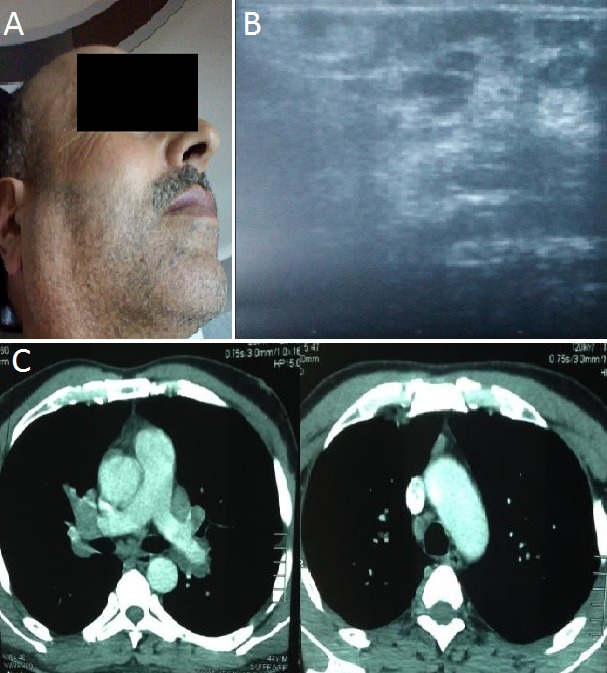
A)tuméfaction de la loge parotidienne; B) échographie cervicale: montre une hypertrophie parotidienne d’échostructure hétérogène en rapport avec de multiples images nodulaires hypoéchogènes de taille variable; C) tomodensitométries thoraciques: (coupes axiales en fenêtre médiastinale) montrent des multiples adénopathies hilaires bilatérales, précarinaire, la loge de barety et médiastinale antérieure non compressible

